# Is there any relationship between the exposure to mycophenolic acid and the clinical status in children with lupus?

**DOI:** 10.1186/1546-0096-9-S1-P248

**Published:** 2011-09-14

**Authors:** C Jurado, B Bader-Meunier, B Ranchin, S Decramer, M Fischbac, E Bérard, F Saint-Marcoux

**Affiliations:** 1 Department of Pharmacology and INSERM UMR –S850, Limoges, France; 2 Departments of Pediatry of: Hôpital Necker, AP-HP, Paris, France; 3 CHU Lyon, France; 4 CHU Toulouse, France; 5 CHU de Strasbourg, France; 6 CHU de Nice, France

## Background

The clinical benefit of Therapeutic Drug Monitoring (TDM) of mycophenolate mofetil (MMF) when used in children with lupus (SLE) has been scarcely studied.

## Aim

(i) To model mycophenolic acid (MPA; the active moiety of MMF) pharmacokinetic profiles (PK); (ii) to explore the relationships between exposure indices to MPA and the clinical status.in paediatric inpatients with SLE receiving a maintenance immunosuppressive therapy including MMF.

## Methods

We launched a non-interventional study with analysis of clinical, biological and pharmacokinetic information. Full-PK profiles of MPA were modelled using an iterative two-stage approach (1). The clinical status was defined by the SLEDAI, the SLE being considered active for a score ≥6. Relationships between MPA through concentrations (C_0_), AUC (Area Under Curve) or AUC/dose values, and the disease’s activity were studied using logistic regression analysis.

## Results

Twenty six children (aged 10 to 17) with SLEDAI score from 0 to 20 (median: 4) followed-up in 5 French centres were included. High PK interpatient variability was observed: AUC_0-12h_=40.51±20.49 mg.h/L. Trough concentrations (C_0_) were poorly correlated to the global exposure to MPA (AUC). Multivariate analysis reported: (i) no relationship between C_0_ and SLEDAI; (ii) patients with an AUC_0-12h_/dose <0.058 h/L were more likely to have an active disease (OR=4.8; 95CI: 0.9-25.0; p=0.067).

## Conclusion

A tendency to a relationship between the lupus activity and the global MPA exposure was observed. Further data are needed to develop PK tools that could estimate the AUC using a limited sampling strategy and to lead prospective trials testing the clinical impact of a MMF TDM based on the AUC.
